# Prior criminal charges and outcomes among individuals initiating office-based buprenorphine treatment

**DOI:** 10.1186/2194-7899-1-2

**Published:** 2013-10-24

**Authors:** Elizabeth E Harris, Janet S Jacapraro, Darius A Rastegar

**Affiliations:** 1 Albert Einstein College of Medicine/Montefiore Medical Center, 1621 Eastchester Road, Bronx, NY 10461 USA; 2 Health Care for the Homeless, 421 Fallsway, Baltimore, MD 21202 USA; 3 Johns Hopkins School of Medicine, Division of Chemical Dependence, Johns Hopkins Bayview Medical Center, 5200 Eastern Avenue, Baltimore, MD 21224 USA

**Keywords:** Opioid-related disorders, Crime, Buprenorphine, Primary health care

## Abstract

**Background:**

There is little data on the impact of prior criminal activity on the treatment of opioid dependence with office-based buprenorphine. The goal of this study was to investigate the association between prior criminal charges and treatment outcomes in a cohort of patients initiating buprenorphine treatment in a primary care practice.

**Methods:**

This was a retrospective study of 252 consecutive patients with opioid dependence who were given at least one prescription for buprenorphine in a primary care practice. A public database was used to collect data on criminal charges prior to enrollment. For every month after initiation of treatment, patients who remained in treatment were classified as “opioid-positive” or “opioid-negative” based on urine drug test results, patient report and clinician assessment. The primary outcomes of interest were treatment retention at one year and achieving ≥ 6 “opioid negative” months.

**Results:**

Most (80%) of the subjects had at least one prior criminal charge. Those with prior criminal charges were more likely to have Medicare or Medicaid insurance, to be unemployed, to use cocaine and to have injected drugs. In the year after initiation of buprenorphine treatment, these subjects had significantly less opioid-negative months than those without criminal charges (5.1 months vs. 6.4 months; p = 0.030), and were less likely to have ≥ 6 opioid-negative months (43.2% vs. 58.4%; p = 0.048). While there was no difference in treatment retention at one-year for those who had any prior history (55.4% vs. 52.0%; p = 0.854), having recent charges (in the previous two years) was associated with poorer treatment retention and drug outcomes. On the other hand, having only distant charges (more than two years prior to treatment initiation) was not associated with poorer outcomes. Using multivariate analysis, recent criminal charges was the only factor significantly associated with treatment retention at one year and achieving ≥ 6 opioid-negative months.

**Conclusions:**

Subjects with recent criminal charges had poorer treatment retention and opioid-abstinence outcomes after initiating office-based buprenorphine treatment. These individuals may benefit from more intensive treatment than is typically offered in a primary care setting.

## Background

Substance dependence is a common medical problem and is associated with criminal activity (Ball et al., 1983). While there is wide variability of rates of substance use disorders in prison populations, the rates are consistently much higher than the general population (Fazal et al., 2006). In a survey by Bureau of the Justice System in 2004, 53% of state and 45% of federal prisoners met criteria for drug dependence or abuse, and 13.1% and 9.2%, respectively, regularly abused opioids, but only 0.3% of state and 0.4% of federal prisoners were managed with opioid replacement therapy (Mumola and Karberg 2006). Given the prevalence of substance use in the criminal justice population and the current lack of access to pharmacologic treatment for opioid dependence, as well as the high rates of recidivism associated with substance use disorders (Hankansson and Berglund, 2012), there is a tremendous need for effective treatments. Incarceration may be an opportunity to initiate treatment for opioid dependence and improve rates of recidivism (Chandler et al., 2009). For prisoners with opioid dependence, methadone maintenance treatment after release from prison has been associated with a reduction in mortality and re-incarceration (Dolan et al., 2005 Larney et al. 2011). Studies suggest that buprenorphine is also a feasible treatment post-release (Springer et al., 2010), treatment retention is associated with a reduction in opiate use and crime (Garcia et al., 2007). and treatment can improve other medical conditions (Springer et al., 2012). Buprenorphine maintenance therapy may be more acceptable than methadone to criminal offenders released from prison (Magura et al., 2009).

While a criminal history may be associated with poorer treatment retention in methadone maintenance therapy (Magura et al., 1998, Villafranca et al., 2006, Kelly et al., 2011, Cox et al., 2012), there is little data on the treatment outcomes associated with office-based buprenorphine; one recent study found that a history of incarceration was not associated with poorer outcomes in office-based buprenorphine treatment (Wang et al., 2010).

The goal of this study is to evaluate the impact of having prior criminal charges on treatment outcomes among patients initiating office-based buprenorphine treatment. We hypothesized that subjects with prior charges would not do as well as those without prior criminal charges and that this association would be strongest for those with recent charges.

## Methods

### Setting

The Comprehensive Care Practice is a primary care clinic on the Johns Hopkins Bayview Medical Center campus, which is staffed by 5 internists, a nurse practitioner, and 3 residents who share a panel of patients. The practitioners provide general primary care, with a focus of serving patients with HIV infection and/or substance use disorders.

Visits for opioid dependence occured as routine primary care visits. There was no uniform protocol or dosing regimen, with buprenorphine doses that ranged from 2 to 32 mg, with most patients on 8–16 mg daily. Patients were typically given a prescription for a one-week supply of buprenorphine initially and induction occurred at home or in the office; follow-up occurred weekly to monthly, based on the provider’s discretion; patients were usually seen more frequently early in treatment or when there was continued substance use. Treatment was continued or discontinued based on the provider’s discretion. The practice did not provide any additional onsite psychosocial services and patients were referred to community resources. A more detailed description of this clinic’s treatment practices can be found in Soeffing et al.,^2009^.

### Subjects

The study included all patients who had been given at least one prescription for sublingual formulation of buprenorphine from August 2003 to September 1, 2007.

### Data collection

As part of a previously performed study (Soeffing et al., 2009), a database of all patients who received at least one prescription for buprenorphine during this period of time had already been created. Data were collected retrospectively from the patient medical records. Demographic information recorded included age, gender, type of insurance and employment status. Substance abuse history collected included substances used and history of injection drug use. Also recorded were relevant comorbidities (hepatitis C, HIV, chronic pain and chronic psychiatric illness).

The time period after receiving the first prescription was divided into twelve monthly blocks for the purposes of this analysis. Patients were considered to be in treatment for each block in which they were prescribed buprenorphine at any point. Patients were considered to be in treatment during the time period covered by their prescriptions and were considered to be out of treatment if there was a gap in the period covered by their prescriptions. For those who dropped out of treatment, they were considered to be in treatment for the duration of time covered by their most recent prescription. There was no fixed protocol for collection of urine drug tests and patients sometimes did not have urine collected when they admitted to recent nonprescribed opioid use or when they had sequential negative urine tests, so for each month in which the patient was receiving treatment, patients were classified as “opioid-positive” or “opioid-negative”. Patients were classified as “opioid-positive” if any of their urine drug tests during that month were positive for opioids (other than those prescribed), they reported using other non-prescribed opioids, or a urine drug test was not collected and their most recent test was positive (practitioners would sometimes not collect urine if the patient reported recent substance use or patients may have left without leaving a urine specimen). Patients were classified as “opioid-negative” if all urine drug tests collected during that month were negative for opioids (other than those prescribed) or if the provider decided not to test the urine and their most recent test was negative.

We utilized the Maryland Judiciary Case Search website (http://casesearch.courts.state.md.us/inquiry/inquiry-index.jsp) to collect data on prior criminal charges. “Criminal charges” are defined in this study as a written accusation charging that an individual named has been accused of committing an act or omitted to do something that is punishable by law. We did not include traffic offenses. This database includes data on all criminal charges in the state of Maryland since 1991. The database provides the defendant name, city and state, date of birth, trial date, charge, and case disposition. We searched this database by each patient’s name and birthdate, and recorded whether the subject had ever had criminal charges prior to initiating treatment. We tabulated the total number of cases during their lifetime and in the two years prior to the date of the first prescription for buprenorphine. We used “cases” as our unit of measurement rather than “charges” because subjects often had multiple individual charges associated with each case. We defined “recent charges” as those within two years of initiation of treatment and “distant charges” as those more than two years prior to initiation of treatment.

### Statistical analysis

The data was analyzed to see if having prior criminal charges was associated with retention and treatment outcomes. We also divided the cohort with prior charges into those with “recent charges” (in the two years prior to the initial visit) and those with only “distant charges” (more than two years prior to the initial visit), as well as those with more than or less than the median number of prior charges. Specific outcomes were retention in treatment, defined as being in treatment 12 months after the initial prescription (including those who may have dropped out temporarily during that year), “opioid-negative” months, and successful treatment, defined as achieving ≥6 “opioid-negative” months in a 12-month period (not necessarily consecutive months).

Bivariate analysis was used to compare demographic factors and outcomes among subjects with and without prior criminal charges. Chi-square tests were used to analyze categorical variables and t tests for continuous variables. P values less than 0.05 were considered statistically significant. Multivariate logistic regression was used to evaluate factors associated with being in treatment at 12 months and achieving ≥6 “opioid-negative” months. Factors that were associated with these outcomes at a p value ≤0.1 were included in the multivariate model; when there was a high collinearity between factors (correlation coefficient >0.4), only one factor was entered into the model. Analysis was performed using PASW software (version 18). This study was approved by the Johns Hopkins Institutional Review Board.

## Results

The study included 252 patients who had been given at least one prescription for sublingual formulation of buprenorphine from August 2003 to September 1, 2007. Three subjects from the original cohort were not included because they could not be identified due to gaps in record-keeping.

### Prior criminal charges and demographic factors

The number of subjects with criminal charges prior to initiating treatment was 202 (80.2%). Among the 202 subjects with prior criminal history, the number of prior cases ranged from 1 to 43 and the median number was 6; 108 (42.9%) subjects had charges in the two years prior to initiation of treatment. Table 1 provides demographic and outcome data on subjects with and without prior criminal charges. A number of characteristics were associated with having prior criminal charges. Subjects with prior charges were more likely to be unemployed or disabled; they were more also more likely to be uninsured or publicly-insured. Those who reported a history of heroin and cocaine use were more likely to have had prior criminal charges, while those who reported prescription drug abuse were less likely to have had prior criminal charges. Hepatitis C virus (HCV) infection and injection drug use (IDU) were also associated with prior criminal charges.

**Table 1:** **Characteristics of subjects with and without prior criminal charges**

Characteristic	Any prior	No prior	***p*** value
	criminal charges	criminal charges	
	(N = 202)	(N = 50)	
**Mean Age (SD)**	40.2 (9.2)	37.9 (10.8)	0.132
**Sex**			
**Male**	112 (55.4%)	30 (60.0%)	0.561
**Female**	90 (44.6%)	20 (40.0%)	
**Insurance**			
**Commercial**	71 (35.1%)	33 (66.0%)	<0.001
**Medicaid**	78 (38.6%)	11 (22.0%)	0.028
**Medicare**	47 (23.3%)	4 (8.0%)	0.016
**None**	6 (3.0%)	2 (4.0%)	0.710
**Employment Status**			
**Employed**	75 (37.1%)	38 (76.0%)	<0.001
**Unemployed**	66 (32.7%)	8 (16.0%)	0.020
**Disabled**	61 (30.2%)	4 (8.0%)	0.001
**Abused Substances**			
**Heroin**	176 (87.1%)	33 (66.0%)	<0.001
**Opioid Rx**	47 (23.3%)	25 (50.0%)	<0.001
**Cocaine**	116 (57.4%)	18 (36.0%)	0.007
**Alcohol**	32 (15.8%)	10 (20.0%)	0.480
**Benzodiazepines**	18 (8.9%)	5 (10.0%)	0.811
**IDU**	128 (63.4%)	24 (48.0%)	0.047
**Co-morbidities**			
**HIV**	29 (14.4%)	7 (14.0%)	0.949
**HCV**	112 (55.4%)	13 (26.0%)	<0.001
**Psychiatric**	103 (51.0%)	22 (44.0%)	0.376
**Chronic Pain**	35 (17.3%)	11 (22.0%)	0.444

Opioid Rx: prescription opioids, IDU: injection drug use, HIV: human immunodeficiency syndrome, HCV: hepatitis C antibody positive.

### Association between criminal charges and treatment outcomes

The association between prior criminal charges and the main treatment outcomes is summarized in table 2. Overall, subjects with prior criminal charges were as likely to be in treatment at 12 months as those without (55.4% vs. 52.0%; p = 0.854). However, subjects with criminal charges were less likely to achieve ≥6 opioid negative months (43.1% vs. 60.0%; p = 0.032) and had significantly fewer opioid-negative months than those without prior criminal charges (mean 5.1 months vs. 6.7 months; p = 0.028).

**Table 2:** **Relationship between prior criminal charges, treatment retention and drug treatment outcomes**

Prior charges	In treatment at 12	Mean number of opioid-	≥6 opioid-negative months
	months (%)	negative months	(%)
None	27 (52.0%)	6.68	30 (60.0%)
(*n* = 50)			
Any charges	112 (55.4%)	5.12	87 (43.1%)
(*n* = 202)	*p* = 0.854^a^	*p* = 0.028^a^	*p* = 0.032^a^
1-6 cases	57 (54.3%)	5.36	47 (44.8%)
(*n* = 105 )	*p* = 0.973^a^	*p* = 0.103^a^	*p* = 0.076^a^
≥7 cases	55 (56.7%)	4.87	40 (41.2%)
(*n* = 97)	*p* = 0.755^a^	*p* = 0.015^a^	*p* = 0.031^a^
Recent charges	58 (53.7)	4.50	40 (37.0%)
(n = 108)	*p* = 0.014^b^	*p* = 0.004^b^	*p* = 0.010^b^
Distant charges	62 (66.0%)	5.84	47 (50.0%)
(n = 94)	*p* =0.160^a^/0.005^c^	*p* = 0.252^a^/0.031^c^	*p* = 0.252^a^/0.063^c^

“Recent charges” are those that occurred in the 2 years prior to treatment initiation, “distant charges” occurred more than 2 years prior to treatment iniation.

^a^*p* value for difference with subjects with no prior charges.

^b^*p* value for difference with subjects with no recent charges.

^c^p value for difference with subjects with recent charges.

There appeared to be a dose–response relationship between number of prior cases and outcomes; subjects with six or fewer prior cases (at or below the median) did not have significantly poorer outcomes than those with no prior history, while those with seven or more prior cases had significantly fewer opioid-negative months and were less likely to achieve ≥6 opioid negative months. Both groups were as likely to remain in treatment as those with no prior charges.

As shown in Table 2, 108 (42.9%) had criminal charges within two years of initiating treatment. Recent charges appeared to have the most significant association with treatment outcomes. When compared to those who did not have recent charges (including those with no prior charges), subjects with charges in the two years prior to initiation of treatment were significantly less likely to remain in treatment or to achieve ≥6 opioid negative months and had significantly fewer opioid-negative months. On the other hand, those with only distant charges did significantly better than those with recent charges and their outcomes were not significantly different from those with no prior charges.

Bivariate and multivariate analysis of factors associated with remaining in treatment at 12 months and achieving ≥6 opioid negative months is shown in Table 3. Subjects age 40 or less and those with recent charges were less likely to remain in treatment, while those with prior cocaine use were more likely. In multivariate analysis, cocaine use was significantly associated with remaining in treatment at 12 months, while having recent charges was associated with a lower likelihood of remaining in treatment. For the outcome of achieving ≥6 opioid negative months, subjects with recent charges were less likely to achieve this outcome, as were those who used heroin. On multivariate analysis, both heroin use and recent charges were significantly associated with a lower probability of achieving ≥6 opioid negative months. Recent criminal charges was the only factor that was significantly associated with both outcomes.

**Table 3:** **Bivariate and multivariable analysis of factors associated with being in treatment in 12 months and achieving ≥6 opioid negative months**

Variable	In treatment at 12 months		≥6 opioid negative months	
	OR (CI)	AOR (CI)	OR (CI)	AOR (CI)
Age ≤ 40	**0.58 (0.36 - 0.97)**	0.61 (0.37 – 1.02)	0.96 (0.59 – 1.58)	
	***p*** **= 0.036**	*p* = 0.062	*p* = 0.960	
Female	0.69 (0.42 - 1.14)		1.00 (0.60 – 1.64)	
	*p* = 0.148		*p* = 0.985	
Employed	1.04 (0.63 - 1.72)		1.43 (0.87 – 2.36)	
	*p* = 0.864		*p* = 0.160	
Heroin abuse	0.97 (0.50 – 1.88)		**0.40 (0.20 – 0.79)**	**0.43 (0.21 – 0.86)**
	*p* = 0.924		***p*** **= 0.008**	***p*** **= 0.016**
Cocaine abuse	**1.80 (1.09 – 2.98)**	**1.75 (1.05 – 2.92)**	1.20 (0.73 – 1.97)	
	***p*** **= 0.022**	***p*** **= 0.032**	*p* = 0.481	
Recent Charges	**0.53 (0.32 – 0.88)**	**0.54 (0.32 – 0.90)**	**0.51 (0.31 – 0.85)**	**0.54 (0.32 – 0.91)**
(past 2 years)	***p*** **= 0.015**	***p*** **= 0.019**	***p*** **= 0.010**	***p*** **= 0.021**

Statistically significant associations are shown in bold. The following factors were excluded due to high correlation with another factor (indicated in parenthesis): commercial insurance (employed), prescription opioid abuse (heroin use), injection drug use (heroin use), any prior charges and ≥ 7 prior cases (recent charges).

## Conclusions

In summary, we found that 80% of patients with opioid dependence initiating buprenorphine maintenance therapy in this primary care setting had prior criminal charges. While a history of criminal charges did not negatively affect treatment retention, those with prior charges had significantly fewer opioid-negative months and were less likely to have ≥6 opioid-negative months. Those with more prior criminal cases or recent charges did worse than those with fewer or more distant charges.

Our findings are consistent with a number of studies conducted with subjects on methadone maintenance. For example, Cox and colleagues (2011) reported that having six or more lifetime arrests was associated with an approximately 3-fold increase in risk of voluntary or involuntary discharge from a methadone program. Those awaiting charges, trial or sentence likewise had a higher risk of voluntary or involuntary discharge. In a previous analysis, we found that prior charges, particularly recent charges, were highly correlated with subsequent charges (Harris et al., 2012); it is possible that some of the poorer outcomes could have simply been due to incarceration.

Our finding that having any prior criminal charges was not associated with poorer treatment retention is consistent with a recent study of a cohort of subjects receiving office-based buprenorphine treatment, which found that a history of incarceration was not associated with treatment retention (Wang et al., 2010). However, Wang et al. reported that a history of incarceration was likewise not associated with increased drug use after initiation of office-based treatment, while we found poorer treatment outcomes among those with a history of criminal charges, particularly those with recent charges. The disparity in our findings may be due to one or more of the differences between the two studies. Unlike our study, Wang et al. excluded individuals who were dependent on other substances, or if they did not complete the 2-week induction period, thus perhaps selecting for patients who would tend to do better in treatment. Additionally, the subjects in their study received more intensive counseling and monitoring than that provided in our primary care setting. Moreover, many of their subjects who reported never being incarcerated had a criminal history and they did not distinguish between recent incarceration and incarceration in the distant past; our findings suggest that criminal charges may be a better predictive factor, particularly when the charges have occurred recently.

Our findings may be relevant to the development of treatment strategies for recently-incarcerated persons who are opioid dependent. While we did not look at incarceration per se, our findings suggest that recent criminal activity is associated with poorer outcomes. These individuals may benefit from more intensive treatment and support than can be offered in a typical primary care practice. While many may express a preference for office-based buprenorphine treatment, it is possible that they would do better in a program (for example, a traditional methadone maintenance program) that offers closer supervision and more psychosocial support than is typically available in a primary care setting. On the other hand, previous studies suggest that buprenophine maintenance is effective for recently incarcerated individuals (Garcia et al., Magura et al., 2009) and may be preferred over methadone by incarcerated individuals (Awgu et al., 2010). It should be noted that in our study, many of those with recent criminal activity did stay in treatment (over 50% at 12 months) and many were abstinent from opioids.

A major limitation of our study is that we looked only at criminal charges; not all criminal activity leads to criminal charges and not all charges are necessarily indicative of criminal activity (i.e., some may have been wrongly charged). On the other hand, criminal charges are an objective measure and likely correlate with criminal activity. Another limitation is that we looked at the outcomes of a cohort treated at a single practice in Baltimore; this may not reflect the outcomes of subjects treated at other sites or in other localities. We also relied on chart review and fairly infrequent drug testing for our outcome measures. A final limitation is that we only looked at criminal charges recorded in Maryland, and could not include charges in other states. On the other hand, unlike many other studies, we used an “intention-to-treat” model and included everyone who had received at least one prescription for buprenorphine, not just those who remained in treatment; so our findings are probably more representative of the outcomes of all who enter treatment, not just those who remain in treatment. Also, we used an objective measure, rather than patient report.

In summary, our study found that subjects with criminal charges tended to do worse than those with no prior charges, but the difference appears to be limited to those with recent criminal charges. Subjects with distant prior charges (none in the two years before initiation of treatment) did as well as those with no prior charges. Although some of the individuals with recent criminal charges appeared to do well with office-based buprenorphine treatment, many may benefit from more intensive treatment and monitoring than can be provided in a typical primary care setting. Further research is needed to determine the most effective treatment approach for these individuals.
